# Robust Optimal Control Design for Performance Enhancement of PWM Voltage Source Inverter

**DOI:** 10.3390/mi13030435

**Published:** 2022-03-12

**Authors:** En-Chih Chang, Hung-Liang Cheng, Chien-Hsuan Chang, Rong-Ching Wu, Chun-An Cheng, Zheng-Kai Xiao, Wen-Jie Lu, Zhi-Yu Wei

**Affiliations:** Department of Electrical Engineering, I-Shou University, No.1, Sec. 1, Syuecheng Rd., Dashu District, Kaohsiung 84001, Taiwan; enchihchang@isu.edu.tw (E.-C.C.); hlcheng@isu.edu.tw (H.-L.C.); chchang@isu.edu.tw (C.-H.C.); rcwu@isu.edu.tw (R.-C.W.); a5690036@gmail.com (Z.-K.X.); enchanted12041015@gmail.com (W.-J.L.); smoac14698@gmail.com (Z.-Y.W.)

**Keywords:** enhanced restrictive sliding mode control, density particle swarm optimization, PWM voltage source inverter

## Abstract

PWM (pulse-width modulation) voltage source inverters are used in a wide range of AC power systems where the output voltage must be controlled to follow a sinusoidal reference waveform. In order to achieve precision and fast-tracking control, restrictive sliding mode control (RSMC) provides a fast system state convergence time. However, the RSMC still suffers from the chattering problem, which leads to high harmonic distortion and slow response of the inverter output state. Furthermore, the load of the inverter may be severe load changing and the control parameters become difficult to adjust, worsening the adaptability to achieve the desired control of the inverter output. In this paper, a robust optimal control design comprised of an enhanced restrictive sliding mode control (ERSMC) and density particle swarm optimization (DPSO) algorithm is proposed, and then applied to PWM voltage source inverters. The ERSMC not only has finite time convergence but also provides chatter elimination. The DPSO is highly adaptable for acquiring the control parameters of the ERSMC and finding the best solution in the global domain. The proposed controller is realized for the actual PWM voltage source inverter controlled by a TI DSP-based development platform, so that the inverter output voltage has fast dynamic response and satisfactory steady-state behavior despite high load changing and non-linear disturbances.

## 1. Introduction

With the increasing demand for high quality power supplies, PWM voltage source inverters have become an important unit of high-performance power conversion systems such as solar energy systems, wind energy systems and fuel cell systems [1,2,3,4]. High-performance PWM voltage source inverters must provide high quality AC output voltages, even at high load changing/non-linear loading, and feedback control technology can achieve such a goal. The performance of the inverter is usually evaluated according to the following criteria: (1) The presence of a low total harmonic distortion (THD) output voltage waveform under linear or non-linear loading conditions. (2) The existence of rapid dynamic response to step changes in load. (3) The steady-state error should be as small as possible. In order to obtain high quality output voltages from inverters, many control methods have been proposed, such as boundary control, repetitive control and H-infinity scheme [5,6,7,8]. Although the above methods are well within the control of inverters, they are either time-consuming or cumbersome to calculate. In addition, proportional-integral-differential (PID) controllers were one of the first control methods to be developed and are widely used in the industry due to their simple control structure and design. However, the load of PWM voltage source inverters is variable, which can be a sudden load change. This means that the output voltage of an inverter is not a perfect sine wave and exists in harmonic distortion waveform with poor recovery of the transient voltage. Even if advanced PID controllers are developed to improve the above-mentioned shortcomings of conventional PIDs, the control parameters are not easy to obtain, and the entire control parameters determination often needs to be combined with optimization algorithms. Sliding mode control (SMC) is known to be highly resistant to parameter uncertainties and external disturbances. Many published works on SMC have also been applied to the control of inverters [9,10,11]; however, these works use conventional sliding mode control with linear sliding surfaces, which are characterized by a low-order system when the desired sliding surface is constrained, and then the system trajectory slides along the sliding surface to the origin, i.e., there is an infinitely long system state convergence time. In recent years, restrictive sliding mode control (RSMC) has been developed for non-linear systems, offering faster convergence mechanism than conventional SMC with linear sliding surfaces. Based on the fast convergence properties of the terminal attractor, the RSMC can bring the system tracking error to zero in a restricted time and then establish more accurate control, which is suitable for the control of PWM voltage source inverters [12,13,14]. However, the RSMC described above still suffers from chatter problem. This problem is one of the shortcomings of the restrictive sliding mode control implementation. The chatter problem can provoke unmodelled high frequency-controlled plant dynamics; sometimes such a problem can even cause system instability. Several methods have been proposed to improve the chatter problem, such as the iterative learning methodology and the adaptive control scheme. Although these methods have improved the chatter problem as well as the robustness of their transient behaviour in the presence of external disturbances and unmodelled dynamics, they are either computationally time-consuming or require very complex calculations [15,16]. In order to solve the above problem, this paper uses the ERSMC to design the control law of the single-phase voltage source inverter, and a density particle swarm optimization (DPSO) algorithm to automatically adjust the control parameters of the ERSMC to gain the optimal solution. Density particle swarm optimization (DPSO) algorithm is based on the concept of the probability of density selection in the immune algorithm being introduced into the PSO algorithm [17,18,19,20]. The DPSO selectively guides the update of particles based on the magnitude of the density, thus speeding up the convergence of the algorithm. It has easy calculation and enhanced population diversity that demonstrates global seeking ability and has been widely used to solve many optimization problems [21,22,23,24]. It can improve the drawback of traditional PSO algorithm which tends to prematurely converge into local extrema. The DPSO can find optimal values for ERSMC control parameters, therefore significantly improving control performance and avoiding complicated testing and trial-and-error adjustments of parameters. The controller concept is simple to understand, computationally efficient and easily convertible, resulting in more precise and fast-tracking control. Whether the load is linear or highly non-linear, the proposed controller will enable fast dynamics and steady-state performance in the output voltage of PWM voltage source inverters. The PWM voltage source inverter is also compared with a PWM voltage source inverter controlled by a conventional RSMC, showing the superior performance of the proposed inverter.

## 2. Modeling of PWM Voltage Source Inverter

**Figure 1:**
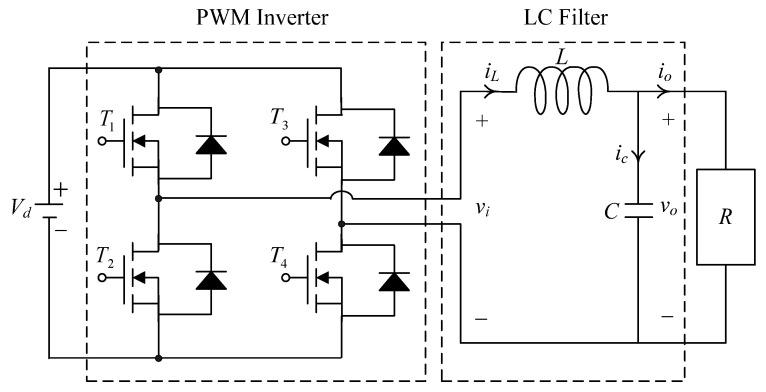
Circuit of PWM voltage source inverter.

Figure 1 illustrates the circuit diagram of a PWM voltage source inverter followed by an LC filter. The PWM voltage source inverter feeds the DC input into a series of PWM pulses based on a modulation signal. The function of the second order LC filter is to remove the high frequency component of the chopped output voltage, $v_{i}$. The DC supply is considered to be the ideal constant voltage supply. As shown in Figure 1, the state equations of PWM voltage source inverter with an LC filter and a resistance R can be described by Kirchhoff’s voltage and current laws:

(1) $$\begin{array}{c} L{\dot{i}}_{L}+v_{o}=v_{i}\\ i_{L}=C{\dot{v}}_{o}+\frac{v_{o}}{R} \end{array}\}$$

From the (1), the dynamic equation yields

(2) $${\ddot{v}}_{o}+\frac{1}{RC}{\dot{v}}_{o}+\frac{1}{LC}v_{o}=\frac{K_{PWM}}{LC}u$$

where u represents the control signal, and $v_{i}$ is the product of u and inverter proportional gain $K_{PWM}$ by the methodology of state-space averaging.

**Figure 2:**
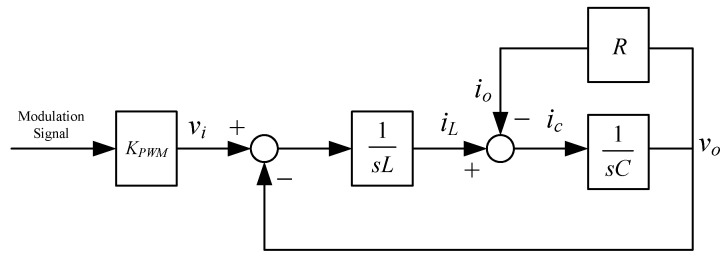
Linear model of PWM voltage source inverter.

Assuming that the switching frequency is high enough to ignore the dynamics of the inverter, i.e., that the inverter can be modeled as a constant gain, $K_{PWM}$. The inverter, filter and load can be transformed into the block diagram shown in Figure 2.

**Figure 3:**
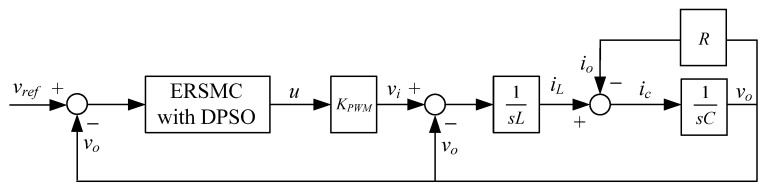
Block diagram of the PWM voltage source inverter with the proposed controller.

The block diagram of a PWM voltage source inverter with the proposed controller is shown in Figure 3. This method senses the voltage in the capacitor and uses the sensing signal to control the voltage feedback loop to ensure a sinusoidal output voltage.

Defining $e_{1}=v_{ref}-v_{o}$ and $e_{2}={\dot{e}}_{1}$, and then from the Figure 3, the error differential equations expressing the system can be obtained as

(3) $$\begin{array}{c} {\dot{e}}_{1}=e_{2}\\ {\dot{e}}_{2}=-a_{1}e_{1}-a_{2}e_{2}-bu+f \end{array}\}$$

where $a_{1}=\frac{1}{LC}$, $a_{2}=\frac{1}{RC}$, $b=\frac{K_{PWM}}{LC}$, $f=a_{1}v_{ref}+a_{2}{\dot{v}}_{ref}+{\ddot{v}}_{ref}$ is regarded as a disturbance limiting to ‖f‖<δ and δ>0. The parameter $e_{1}$ acts as a measurement of change from $v_{ref}$ to $v_{o}$. The system (3) will become stable where $e_{1}$ eventually converges to zero with the control signal u being completely designed so that the output of the inverter is kept at the same level as the desired $v_{ref}$.

## 3. Control Design

This section begins with a review on the problem of nonlinear systems based on the conventional RSMC, and then proceeds to design the proposed controller. Take into account a second-order nonlinear equation given below.

(4) $$\begin{array}{c} {\dot{\chi }}_{1}=\chi_{2}\\ {\dot{\chi }}_{2}=g(\chi_{1},\;\chi_{2})+hu+N(\chi_{1},\;\chi_{2},\;t) \end{array}\}$$

where $\chi_{1}$ and $\chi_{2}$ denote the state variables, $g(\chi_{1},\;\chi_{2})$ signifies smooth nonlinear function, h represents the control gain, u is the control input, and $N(\chi_{1},\;\chi_{2},\;t)$ indicates the uncertainties, in which it is taken to be constrained by $\left|N(\chi_{1},\;\chi_{2},\;t)\right|<\bar{U}$ with $\bar{U}$ being a positive constant.

The sliding surface for the conventional RSMC can be composed as

(5) $$\sigma =\chi_{2}+\eta \chi_{1}^{\kappa }$$

or

(6) $$\sigma =\chi_{2}+\mu \chi_{1}+\eta \chi_{1}^{\kappa }$$

where μ and η symbol positive real numbers, and 0<κ<1. From the (5) and (6) implies that there will be a convergence of system state $\chi_{1}$ towards the equilibrium with restrictive time, as well as a similar convergence of system state $\chi_{2}$ into the equilibrium with restrictive time. With $\chi_{1}$ and $\chi_{2}$ concentrated to the equilibrium is restrictive time, the σ both in the (5) and (6) conforms to the sliding mode σ=0 at a restrictive time as well. With the example of the (5), its dynamics during the sliding mode can be formulated as ${\dot{\chi }}_{1}=\chi_{2}=-\eta \chi_{1}^{\kappa }$. The dynamic behavior becomes restrictive time attainable to $\chi_{1}=0$ by the relaxation time $t_{r}={\left|\chi_{1}(0)\right|}^{1-\kappa }/\eta (1-\kappa )$ for the starting state $\chi_{1}(0)\neq 0$ at t=0 being granted. Then the Jacobian matrix ${J|}_{\chi_{1}\to 0^{+}}=\partial {\dot{\chi }}_{1}/\partial \chi_{1}$ along with one eigenvalue of its first order approximation matrix is determined to have minus infinity. The state of the system leads to restrictive time attainability by converging to the equilibrium at an infinite rate caused by the infinite minus values of the eigenvalues. However, the following problems should be pointed out: (i) In the case of 0<κ<1, there will be an imaginary number $\chi_{1}^{\kappa -1}$. (ii) If $\chi_{2}$ does not equal to zero while $\chi_{1}$ waits for zero and 0<κ<1, a singularity is induced by $\chi_{1}^{\kappa -1}\chi_{2}$ in the control law. In this case, it gives rise to an unrestricted control behavior, which makes the system uncontrolled.

To address the issue of not real numbers in the (5) and (6), it is necessary to consider conventional fast RSMC below:

(7) $$\sigma =\chi_{2}+\eta {\left|\chi_{1}\right|}^{\kappa }\mathrm{sgn}(\chi_{1})$$

or

(8) $$\sigma =\chi_{2}+\mu \chi_{1}+\eta {\left|\chi_{1}\right|}^{\kappa }\mathrm{sgn}(\chi_{1})$$

where sgn(⋅) is a signum function. There are still singularities occurring despite the fact that the (7) and (8) have tackled the imaginary number effect in the control law, together with the restrictive time attainability.

The ERSMC sliding surface in response to the error state Equation (3) to secure convergence quickly with no singularity occurring for a restricted time could be expressed as

(9) $$\sigma =e_{1}+\alpha_{1}^{-1}{\left\|e_{1}\right\|}^{m+1}+\alpha_{2}^{-1}e_{2}^{\gamma }$$

where m>0, $\alpha_{1}>0$, $\alpha_{2}>0$ and 1<γ<2. Next, a proportional-differential power reaching law is suggested as

(10) $$\dot{\sigma }=-k_{p}(\sigma^{\xi }+\mathrm{sgn}(\cdot )k_{p})-k_{d}\dot{\sigma }=-k_{p}{\left(1+k_{d}\right)}^{-1}(\sigma^{\xi }+\mathrm{sgn}(\cdot )k_{p})$$

where $k_{p}>0$, $k_{d}>0$, ξ<2, and Select continuous function tanh(σ/ρ) carrying the appropriate positive number ρ instead of the sgn function to reduce the chatter.

Following the procedure in (9) and (10), a control law u produces

(11) $$u=b^{-1}\{[-k_{e}e-\alpha_{2}\gamma^{-1}e_{2}^{2-\gamma }(1+(m+1){\left\|e_{1}\right\|}^{m}/\alpha_{1})]+k_{p}{\left(1+k_{d}\right)}^{-1}(\sigma^{\xi }+\mathrm{sgn}(\cdot )k_{p})\}$$

where $k_{e}$ represents the feedback gain of equivalent control, which creates the desired sliding mode while system uncertainties equal to zero. The (11) leads to the system state that will be compelled towards σ=0, which converges in a finite amount of time.

Proof: The definition of a Lyapunov candidate gives the following:

(12) $$V=\frac{1}{2}\sigma^{2}$$

Based on the dynamical system trajectory along from the control law (11) with the use of the Lyapunov candidate, time derivative of the V becomes

(13) $$\begin{array}{cc} \dot{V} & =\sigma \dot{\sigma }\\ & \leq -\sigma \cdot [k_{p}{\left(1+k_{d}\right)}^{-1}(\sigma^{\xi }+\mathrm{sgn}(\cdot )k_{p})] \end{array}$$

When σ≠0, $e_{2}\neq 0$ is valid, therefore $\dot{V}\leq 0$ holds. Under the condition $e_{2}\neq 0$, it is proven that the system fulfils the Lyapunov stability condition which allows it to quickly arrive at the sliding surface within a restricted time. Yet, if drastic variations or great nonlinearities in the system load, which prevent the eventual system output from following the reference sine wave, rendering the tracking behavior imprecise. Aiming to acquire the global best solution, the DPSO algorithm can be used. The (14) and (15) represent the model of particle evolution, which can then update the velocity and location of each particle as it flies to its target.

(14) $$\upsilon_{j+1}=\omega \upsilon +p_{1}ram_{1}(X_{pb,\;j}-X_{j})+p_{2}ram_{2}(X_{gb,\;j}-X_{j})$$

(15) $$X_{j+1}=X_{j}+\upsilon_{j+1}$$

where $\upsilon_{j+1}$ represents present flying speed, $X_{j}$ indicates present position, $\chi_{pb}$ shows individual best position, $X_{gb}$ stands for global best position, $p_{1}$ and $p_{2}$ signify learning factor, $ram_{1}$ and $ram_{2}$ are random number amidst 0 and 1, and ω denotes inertia weight. The artificial immune algorithm specifies the meaning of density, which corresponds to solving optimization problems. The best solution fulfilling the constraint refers to the antigen; the candidate solution belongs to the antibody. The compatibility between antibody and antigen depends on whether the candidate solution meets the optimal solution. Thus, the density of the n-th particle can be expressed as [25,26,27].

(16) $$d(x_{n})=1/\sum_{z=1}^{M}\left|\Gamma (x_{n})-\Gamma (x_{z})\right|$$

From the (16), the probability selection based on particle density can be obtained as

(17) $$ℜ(x_{n})=\sum_{z=1}^{M}\left|\Gamma (x_{n})-\Gamma (x_{z})\right|/\sum_{n=1}^{M}\sum_{z=1}^{M}\left|\Gamma (x_{n})-\Gamma (x_{z})\right|$$

where $x_{n}$ and $\Gamma (x_{n})$ denote the n-th particle and its fitness function, respectively. The step procedure of the DPSO algorithm can be summarized in the following. Step 1: The parameters can be initialized to find the size of the population, largest iterative number, and factors of learning. Step 2: The initial position and velocity of each particle are stochastically generated. Step 3: The fitness of each particle can be estimated with respect to the objective function. Step 4: By comparing the adaptation of each particle with the individual extremum experienced by the particle. In the case of favorable adaptation, the position is considered as the current best position, followed by a comparison with the global extremum experienced by the entire particle swarm; in the case of favorable adaptation, the global best position should be renewed. Step 5: Based on the velocity and position shown in (14) and (15), the particle velocity and position have to be renewed. Step 6: The concentration and probability of particles can be determined in accordance with (16) and (17) once there is a stagnant population evolution. Step 7: Evaluate the renewed particle adaptation as well as update the own best solution and the global best position. Step 8: Judge when the maximum number of iterations has been completed. It is repeated from step 4 to 6 and output the global best solution. Based on Equation (17), if there are more antibodies with similarity to antibody n, the probability of antibody n being picked decreases; inversely, when fewer antibodies are similar to antibody n, the probability of antibody n being chosen increases. Such a mechanism permits the evolution with low adaptation individuals. Consequently, a probabilistic selection procedure on the basis of the density of antibodies provides for the diversity of antibodies.

## 4. Results and Analysis

**Figure 4:**
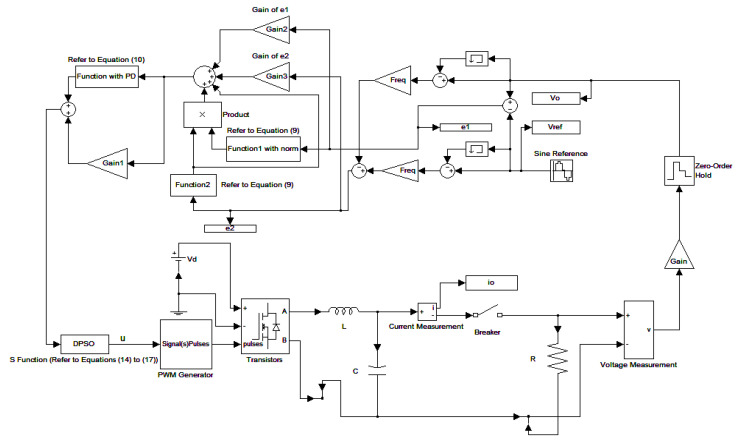
Graphical form of control signal conversion in Simulink/Matlab environment.

**Figure 5:**
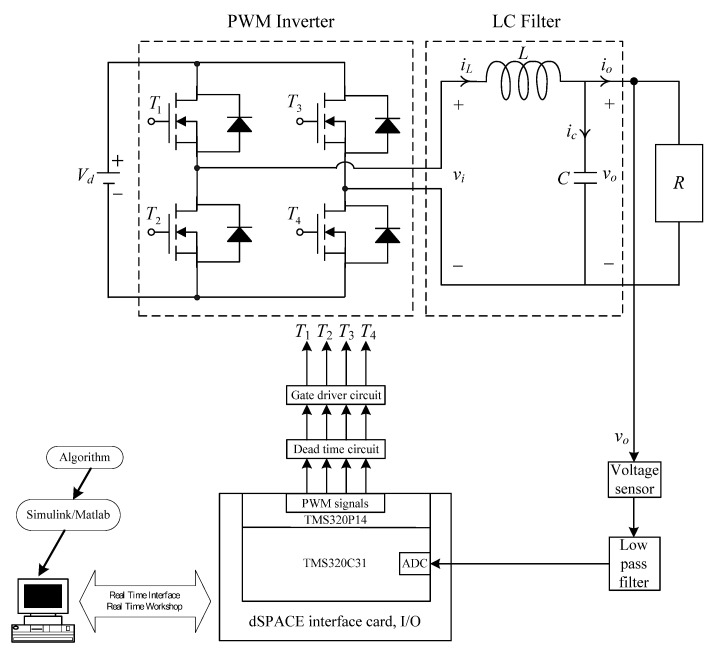
Block diagram of the experimentation.

**Figure 6:**
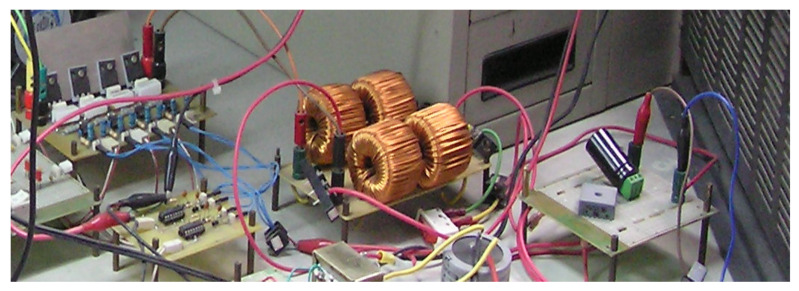
Photograph of the hardware implementation.

**Figure 7:**
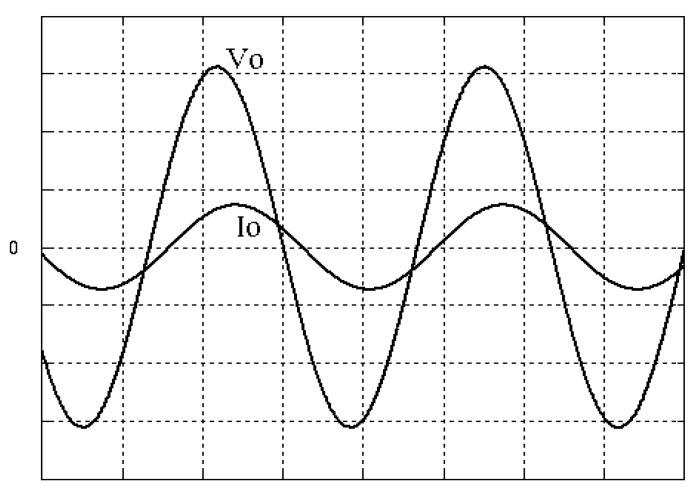
Simulated output waveforms with the proposed controller at inductive load (vertical: 50 V/div and 20 A/div; horizontal: 5 ms/div).

**Figure 8:**
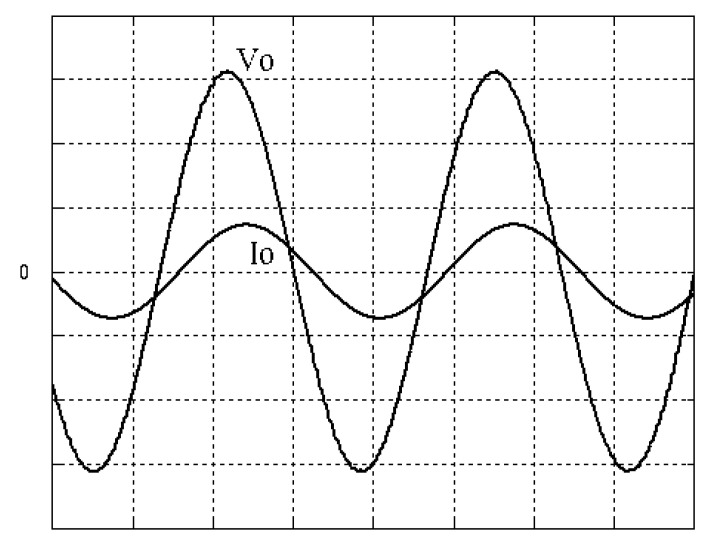
Simulated output waveforms with conventional RSMC at inductive load (vertical: 100 V/div and 20 A/div; horizontal: 5 ms/div).

**Figure 9:**
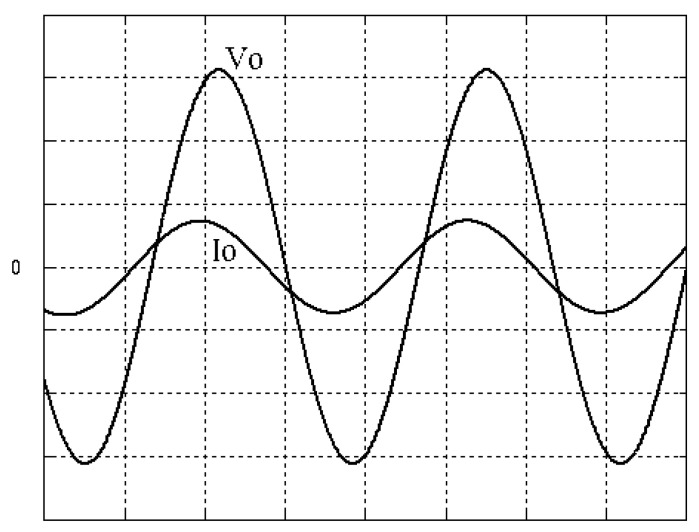
Simulated output waveforms with the proposed controller at capacitive load (vertical: 50 V/div and 20 A/div; horizontal: 5 ms/div).

**Figure 10:**
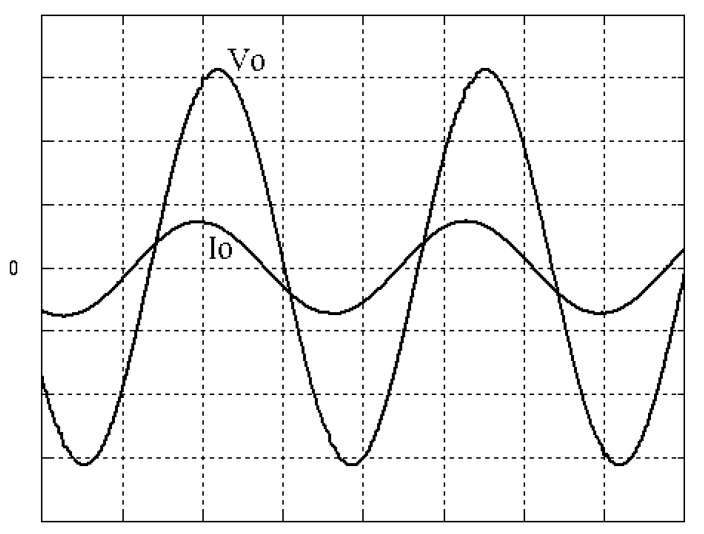
Simulated output waveforms with conventional RSMC at capacitive load (vertical: 100 V/div and 20 A/div; horizontal: 5 ms/div).

**Figure 11:**
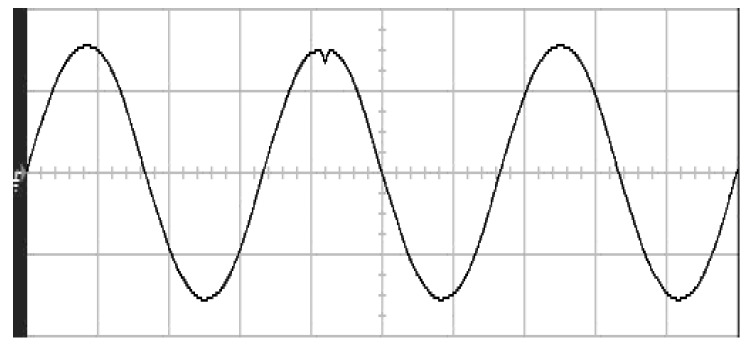
Experimental output voltage with the proposed controller under step changing in resistive load (vertical: 100 V/div; horizontal: 5 ms/div).

**Figure 12:**
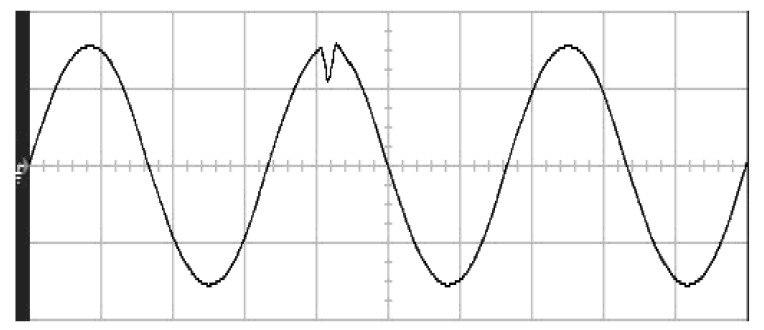
Experimental output voltage with conventional RSMC under step changing in resistive load (vertical: 100 V/div; horizontal: 5 ms/div).

**Figure 13:**
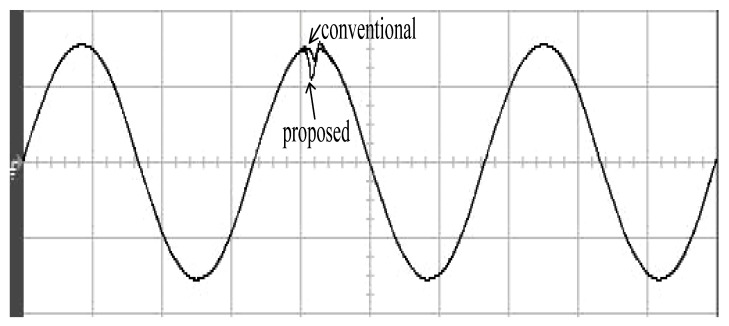
Comparison of experimental output voltage with the proposed controller and the conventional RSMC under step changing in resistive load (vertical: 100 V/div; horizontal: 5 ms/div).

**Figure 14:**
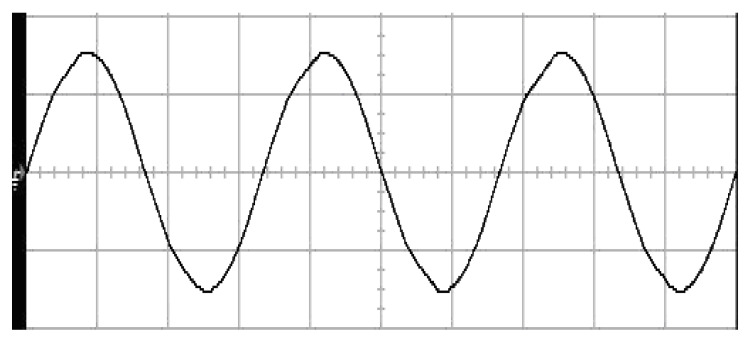
Experimental output voltage with the proposed controller at nonlinear load (vertical: 100 V/div; horizontal: 5 ms/div).

**Figure 15:**
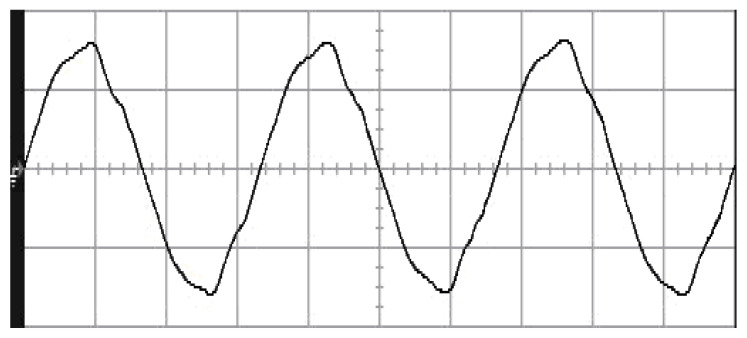
Experimental output voltage with conventional RSMC at nonlinear load (vertical: 100 V/div; horizontal: 5 ms/div).

The parameters of the inverter used for the proposed method are listed in Table 1. For the LC filter in the PWM voltage source inverter, it is sufficient to determine values of components in accordance with the suggestions given in [28,29,30]: (i) By making the decision on switching frequency [29,30]. There is a requirement to pick a high adequate switching frequency aiming to minimize filter size, typically ranging from twenty kHz to forty kHz for MOSFET (metal-oxide-semiconductor field-effect transistor switches). (ii) A parameter dependent on an LC filter’s cut-off frequency can be assigned [28]. With a small parameter, there is a major decay and a minor scaling at the switching frequency as well as at the fundamental frequency. One can derive a minimization of the parameter along with a value of modulation under 0.95 ideally. (iii) It is important to consider a parameter dependent on the switching frequency along with the inductance ripple current [28]. The inductance ripple current would be advisable to be among twenty percent to forty percent. It is then computed the values of L and C by taking the chosen parameters from (8), (20), (25) and (26) in the [28]. Both the controller and PWM modules are designated in the Simulink/Matlab environment. The real-time workshop is in turn utilized for auto-generation of optimal C code. The control algorithm is allowed to be implemented in the TMS320C31-based DS1102 microprocessor. An ADC (analog to digital converter) serves to sense the capacitor voltage, and four gating signals are created via main bit I/O. Figure 4 plots the graphical form of control signal conversion in the Simulink/Matlab environment. The block diagram of the experimentation is illustrated in Figure 5 and Figure 6, revealing a photograph of its hardware implementation. The load can be of the following types: inductive, capacitive, step change in resistive load or nonlinear. Figure 7 exhibits the simulated output waveforms achieved with the proposed controller at inductive load, and Figure 8 displays the simulated output waveforms achieved with the conventional RSMC at the same loading situation. The simulated output waveforms are shown in Figure 9 using the proposed controller at capacitive load, and Figure 10 illustrates the simulated output waveforms using the conventional RSMC at similar operating condition. For both inductive and capacitive load applications, the output voltages are quite near to sine wave with almost no distortion giving good steady state precision. Nonetheless, there are observable phenomena as follows: in the case of inductive loading, the current lags behind the voltage by ninety degrees, while under capacitive loading, the current exceeds the voltage by ninety degrees. Because the inductor stores magnetic energy, the inductive load may generate transient negative voltages on the implementation, which cause severe damage to the driving circuit. The capacitive load occasionally has a large inrush current which may lead to a drop in input supply voltage and the heating of MOSFETs in practice, thereby impairing other circuits in the system or failing. In the actual implementation, the difficult loading tests are performed, i.e., the transient performance and steady-state response are examined by step load changes and nonlinear loads, respectively. Figure 11 shows the experimental output voltage of the inverter using the proposed controller at trigger angle, changing from no load to full resistive load (R = 12 ohm) and when the capacitance of the LC filter is varied from the nominal value 20 microfarad to the maximum value 30 microfarad. With the proposed controller, the transient voltage sag is recovered within a very short period of time with good voltage-sag compensation capability. Figure 12 shows the experimental output voltage of the inverter controlled by the conventional RSMC at a trigger angle, changing from no load to full resistive load (R = 12 ohm) and when the capacitance of the LC filter is varied from the nominal value 20 microfarad to the maximum value 30 microfarad. The transient voltage sag of the conventional RSMC cannot be speedily restored to the reference sine wave, and there is an oscillation occurring in the waveform. Figure 13 compares the experimental output-voltage waveforms with the proposed controller and the conventional RSMC under step changing in resistive load. In Figure 14, the experimental output voltage of the proposed inverter with the nonlinear load is portrayed. There is a favorable sine waveform at the output voltage. Its voltage %THD yields 0.61%, which is lower than the industrial standard. Figure 15 presents the experimental output voltage of the conventional RSMC inverter with the nonlinear load, resulting in severe distortion with a high voltage %THD of 14.12%. Table 2 gives the experimental output-voltage sag and %THD under step changing in resistive load and nonlinear loads. Thus, it can be observed that by using the proposed controller, the output voltages are highly robust to transient loading and steady-state loading. This implies that the proposed controller outperforms the IEEE (Institute of Electrical and Electronics Engineers) standard 519–1992 recommending a voltage THD of less than 5%. In terms of voltage sag, the proposed controller complies with IEEE standard 1159–1995 advising rms (root mean square) voltage/current sag (typically with voltage-sag values between 0.1 and 0.9 per unit) from 0. 5 cycles to 1 min in duration at power supply frequency.

## 5. Conclusions and Discussion

**Figure 16:**
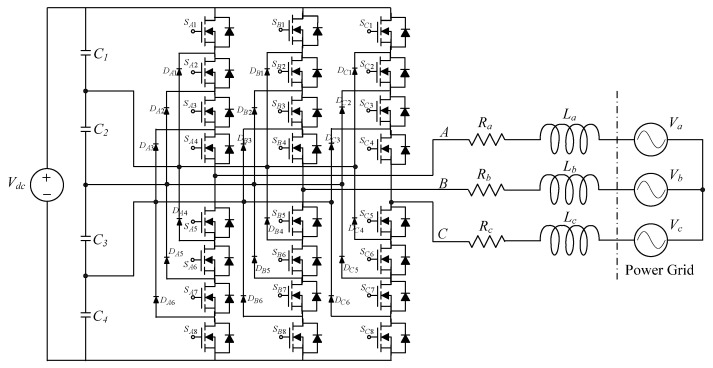
Three-phase neutral-point-clamped five-level inverter using the proposed controller at future research work.

This paper presents a DPSO-based ERSMC applied to voltage source inverters capable of producing fast dynamic performance and good steady-state response. The importance of the ERSMC is the restricted system state converging time. The parameters of the ERSMC should be determined to gain the best performance. Traditionally, these parameters are selected via repeated experiments, which are tedious, difficult to realize and time-consuming. The DPSO is employed to optimize the ERSMC parameters to yield better transience and steady-state behaviors. The Lyapunov’s theorem was used to prove the stability of the proposed method. The restricted time accessibility of the sliding surface, asymptotic stability of the feedback system and fast convergence of the state error were demonstrated. The simulations and experiments with DSP prototype voltage source inverter under transient loading and steady-state loading are shown to verify the applicability of the proposed controller. Noticeably, it is advisable to consider the development of three-phase multilevel inverters for increasing output power [31,32,33,34]. A three-phase three-level inverter is suggested to efficiently manage the upper and lower shoot-through states while it balances the neutral-point voltage with a simple logical circuit. The presented structure delivers a high step-up AC output which supports five levels of voltage [31]. There is a carrier-overlapping PWM introduced for neutral-point-clamped multilevel converter whose capacitive voltages of all DC link under ideal, as well as steady-state circumstances, allow natural balancing [32]. The generalized space vector modulation (SVM) algorithm is adopted to realize a three-phase neutral-point clamped multilevel inverter, where the SVM is accomplished with the idea of nearest level modulation; a gate pulses of the inverter is also guided by a generalized switch matrix, thereby streamlining the algorithm [33]. The neutral-point clamped five-level Inverter with self-balanced switched capacitor is applicable to the grid-tied interfacing of sustainable energies, as well as electric motor drives. The performance has been enhanced with respect to loss of power, common mode voltage, switched stress, as well as output filtering due to the addition in level of output [34]. With the merits as described above, the proposed controller can be incorporated with a high power three-phase neutral-point-clamped five-level inverter, as illustrated in Figure 16, to perform a higher efficient grid-connected inverter at the future research work.

## Figures and Tables

**Table 1:** Parameters of the inverter.

Parameter	Value
$\mathrm{DC}\;\mathrm{supply}\;\mathrm{voltage}\;(V_{d}$)	200 V
$\mathrm{Sin}e\;\mathrm{output}\;\mathrm{voltage}\;(v_{o}$)	110 V_rms_
Frequency of sine output voltage	60 Hz
Nominal filter inductor (L)	0.25 mH
Nominal filter capacitor (C)	20 μF
Resistive load (R)	12 Ω
Switching frequency	30 kHz

**Table 2:** Voltage sag and %THD of output voltage.

**Proposed Controller**		
Experiments	Step changing in load	Nonlinear load
	Voltage sag	%THD
	8.73 Vrms	0.61%
**Conventional RSMC**		
Experiments	Step changing in load	Nonlinear load
	Voltage sag	%THD
	37.12 Vrms	14.12%

## Data Availability

Not applicable.

## References

[1] Wang L., Lam C.S., Wong M.C. (2018). Analysis, Control, and Design of a Hybrid Grid-Connected Inverter for Renewable Energy Generation with Power Quality Conditioning. IEEE Trans. Power Electron..

[2] Yang Z.X., Zeng J.W., Zhang Q., Zhang Z., Winstead V., Yu D.Z. (2021). A Composite Power Decoupling Method for a PV Inverter With Optimized Energy Buffer. IEEE Trans. Ind. Appl..

[3] Soares-Ramos P.P., Oliveira-Assís L.D., Sarrias-Mena R., García-Triviño P., García-Vázquez C.A., Fernández-Ramírez L.A. (2021). Averaged Dynamic Modeling and Control of a Quasi-Z-Source Inverter for Wind Power Applications. IEEE Access.

[4] Kumar M., Tyagi B.A. (2021). Robust Adaptive Decentralized Inverter Voltage Control Approach for Solar PV and Storage-Based Islanded Microgrid. IEEE Trans. Ind. Appl..

[5] He Y., Chung H.S., Ho C.N., Wu W.M. (2017). Direct Current Tracking Using Boundary Control With Second-Order Switching Surface for Three-Phase Three-Wire Grid-Connected Inverter. IEEE Trans. Power Electron..

[6] Julian A.L., Oriti G., Ji C., Zanchetta P. (2018). Single-Phase Energy Management System Operating in Islanding Mode With Repetitive Control and Active Damping. IEEE Trans. Ind. Appl..

[7] Montanari G.C. (2017). Time behavior of partial discharges and life of type II turn insulation specimens under repetitive impulse and sinusoidal waveforms. IEEE Electr. Insul. Mag..

[8] Xia C.Y., Wang W., Chen G.P., Wu X.J., Zhou S.J., Sun Y.J. (2017). Robust Control for the Relay ICPT System Under External Disturbance and Parametric Uncertainty. IEEE Trans. Control. Syst..

[9] Azar A.T., Zhu Q.M. (2015). Advances and Applications in Sliding Mode Control Systems.

[10] Vaidyanathan S., Lien C.H. (2017). Applications of Sliding Mode Control in Science and Engineering.

[11] Utkin V.I. (1977). Variable Structure Systems with Sliding Modes. IEEE Trans. Autom. Contr..

[12] Tabart Q., Vechiu I., Etxeberria A., Bacha S. (2018). Hybrid Energy Storage System Microgrids Integration for Power Quality Improvement Using Four-Leg Three-Level NPC Inverter and Second-Order Sliding Mode Control. IEEE Trans. Ind. Electron..

[13] Rezkallah M., Sharma S.K., Chandra A., Singh B., Rousse D.R. (2017). Lyapunov Function and Sliding Mode Control Approach for the Solar-PV Grid Interface System. IEEE Trans. Ind. Electron..

[14] Zhang Y.J., Wang J., Li H., Zheng T.Q., Lai J.S., Li J.G., Wang J.H., Chen Q. (2020). Dynamic Performance Improving Sliding-Mode Control-Based Feedback Linearization for PV System Under LVRT Condition. IEEE Trans. Power Electron..

[15] Bi G.D., Zhang G.Q., Wang G.L., Wang Q.W., Hu Y.H., Xu D.G. (2021). Adaptive Iterative Learning Control Based Rotor Position Harmonic Error Suppression Method for Sensorless PMSM Drives. IEEE Trans. Ind. Electron..

[16] Li H., Ding X., Xue R.N., Li G.J., Chen Y.M. (2021). Active Damping Adaptive Controller for Grid-Connected Inverter Under Weak Grid. IEEE Access..

[17] Guo W.F., Du H., Cheong J.W., Southwell B.J., Dempster A.G. (2022). GNSS-R Wind Speed Retrieval of Sea Surface Based on Particle Swarm Optimization Algorithm. IEEE Trans. Geosci. Remote Sens..

[18] Song M.M., Liu S.X., Li W.Q., Chen S.Z., Li W.W., Zhang K.K., Yu D.F., Liu L., Liu X.Y. (2021). A Continuous Space Location Model and a Particle Swarm Optimization-Based Heuristic Algorithm for Maximizing the Allocation of Ocean-Moored Buoys. IEEE Access.

[19] Yousaf S., Mughees A., Khan M.G., Amin A.A., Adnan M. (2020). A Comparative Analysis of Various Controller Techniques for Optimal Control of Smart Nano-Grid Using GA and PSO Algorithms. IEEE Access.

[20] Mercangöz B.A. (2021). Applying Particle Swarm Optimization: New Solutions and Cases for Optimized Portfolios.

[21] Alswaitti M., Albughdadi M., Isa A.M. (2018). Density-based particle swarm optimization algorithm for data clustering. Expert Syst. Appl..

[22] Tan Y., Xiao Z.M. Clonal Particle Swarm Optimization and Its Applications. Proceedings of the 2007 IEEE Congress on Evolutionary Computation (CEC 2007).

[23] Ma L.H., Zhang Y., Lu Z.M., Li H. (2013). Research of Particle Filter Based on Immune Particle Swarm Optimization. Inf. Technol. J..

[24] Ge X.C. (2015). The Application of Particle Immune Algorithm in Soccer Equipment Training. MATEC Web Conf..

[25] Du H., Liu D.C., Zhang M.H. (2016). A Hybrid Algorithm Based on Particle Swarm Optimization and Artificial Immune for an Assembly Job Shop Scheduling Problem. Math. Probl. Eng..

[26] Zhang Q.R., Luo M., Wang H.X., Tan J.H. A Hyperlipemia Information Analysis System based on immune algorithm. Proceedings of the 2010 International Conference on Computer Application and System Modeling (ICCASM 2010).

[27] Liu L.P., Jia W.S. (2021). An Intelligent Algorithm for Solving the Efficient Nash Equilibrium of a Single Leader Multi-Follower Game. Mathematics.

[28] Ahmad A.A., Abrishamifar A., Farzi M. A New Design Procedure for Output LC Filter of Single Phase Inverters. Proceedings of the 2010 Power Electronics and Intelligent Transportation System (PEITS).

[29] Dahono P.A., Purwadi A., Qamaruzzaman An LC filter Design Method for Single-Phase PWM Inverters. Proceedings of the 1995 Power Electronics and Drive Systems.

[30] Kim H.S., Sul S.K. (2011). A Novel Filter Design for Output LC Filters of PWM Inverters. J. Power Electron..

[31] Huynh A.T., Ho A.V., Chun T.W. (2020). Three-Phase Embedded Modified-Z-Source Three-Level T-Type Inverters. IEEE Access.

[32] Wang K., Zheng Z.D., Xu L., Li Y.D. (2020). A Generalized Carrier-Overlapped PWM Method for Neutral-Point-Clamped Multilevel Converters. IEEE Trans. Power Electron..

[33] Pratheesh K.J., Jagadanand G., Ramchand R. (2018). A Generalized-Switch-Matrix-Based Space Vector Modulation Technique Using the Nearest Level Modulation Concept for Neutral-Point-Clamped Multilevel Inverters. IEEE Trans. Ind. Electron..

[34] Ye Y.M., Hua T.K., Chen S.J., Wang X.L. (2022). Neutral-Point-Clamped Five-Level Inverter with Self-Balanced Switched Capacitor. IEEE Trans. Ind. Electron..

